# Serological Evaluation of *Mycobacterium ulcerans* Antigens Identified by Comparative Genomics

**DOI:** 10.1371/journal.pntd.0000872

**Published:** 2010-11-02

**Authors:** Sacha J. Pidot, Jessica L. Porter, Laurent Marsollier, Annick Chauty, Florence Migot-Nabias, Cyril Badaut, Angèle Bénard, Marie-Therese Ruf, Torsten Seemann, Paul D. R. Johnson, John K. Davies, Grant A. Jenkin, Gerd Pluschke, Timothy P. Stinear

**Affiliations:** 1 Department of Microbiology, Monash University, Clayton, Victoria, Australia; 2 Department of Microbiology and Immunology, University of Melbourne, Parkville, Victoria, Australia; 3 Groupe d'Étude des Interactions Hôte-Pathogène, Université d'Angers, Angers, France; 4 Centre de Dépistage et de Traitement de l'ulcère de Buruli, Pobè, Benin; 5 Institut de Recherche pour le Développement UMR216, Mère et enfant face aux infections tropicales, Paris, France; 6 Faculté de Pharmacie, Université Paris Descartes, Paris, France; 7 Swiss Tropical Public Health Institute, Basel, Switzerland; 8 Victorian Bioinformatics Consortium, Monash University, Clayton, Victoria, Australia; 9 Department of Infectious Diseases, Austin Health, Heidelberg, Victoria, Australia; Kwame Nkrumah University of Science and Technology (KNUST) School of Medical Sciences, Ghana

## Abstract

A specific and sensitive serodiagnostic test for *Mycobacterium ulcerans* infection would greatly assist the diagnosis of Buruli ulcer and would also facilitate seroepidemiological surveys. By comparative genomics, we identified 45 potential *M. ulcerans* specific proteins, of which we were able to express and purify 33 in *E. coli*. Sera from 30 confirmed Buruli ulcer patients, 24 healthy controls from the same endemic region and 30 healthy controls from a non-endemic region in Benin were screened for antibody responses to these specific proteins by ELISA. Serum IgG responses of Buruli ulcer patients were highly variable, however, seven proteins (MUP045, MUP057, MUL_0513, Hsp65, and the polyketide synthase domains ER, AT propionate, and KR A) showed a significant difference between patient and non-endemic control antibody responses. However, when sera from the healthy control subjects living in the same Buruli ulcer endemic area as the patients were examined, none of the proteins were able to discriminate between these two groups. Nevertheless, six of the seven proteins showed an ability to distinguish people living in an endemic area from those in a non-endemic area with an average sensitivity of 69% and specificity of 88%, suggesting exposure to *M. ulcerans*. Further validation of these six proteins is now underway to assess their suitability for use in Buruli ulcer seroepidemiological studies. Such studies are urgently needed to assist efforts to uncover environmental reservoirs and understand transmission pathways of the *M. ulcerans*.

## Author Summary

Buruli ulcer is a slowly progressive but potentially devastating disease of skin and subcutaneous tissue caused by the bacterium *Mycobacterium ulcerans.* The disease is widespread throughout West and Central Africa, and some countries in the region have established Buruli ulcer control programs. Buruli ulcer is difficult to distinguish from other chronic skin conditions that require different treatments, and there is an urgent need for an accurate point-of-care diagnostic test. In this study, we have used genomic techniques to identify 45 potential *M. ulcerans*–specific antigens, 33 of which we have been able to produce and purify. We tested these proteins against sera from patients, healthy people living in the same region as the patients and from individuals living in a region with no cases of Buruli ulcer. We found that seven proteins were able to elicit antibody responses that were significantly different between patients and the control subjects from the non-endemic region but not from the healthy individuals in the same Buruli ulcer endemic region. Further analysis showed that six of these *M. ulcerans* proteins might be useful as markers of exposure to *M. ulcerans* and could be developed into tools to uncover environmental reservoirs and understand transmission pathways of the bacterium.

## Introduction

*Mycobacterium ulcerans* is the causative agent of the severe necrotizing skin disease known as Buruli ulcer (BU). The clinical presentation of the disease begins with the appearance of a small, painless, nodule or papule. As the infection progresses, necrosis of subcutaneous fat and eventual breakdown of skin occurs leading to the appearance of a characteristic ulcer with an undermined edge [1], [2]. BU has been reported in more than 30 countries world-wide, although the primary burden of disease is carried by those in western and sub-Saharan Africa [3]. Since the 1980s there has been a rapid re-emergence of the disease, where in some endemic regions it is now more common than the two other major mycobacterial diseases, tuberculosis and leprosy [4]. The victims of this disease are most commonly children, although any individual may be affected [5], [6]. Whilst control efforts are underway in many affected countries, a major shortcoming is the lack of a simple, rapid method to confirm infection with *M. ulcerans*.

The necrosis of subcutaneous fat seen in BU is due to the actions of a family of polyketide toxins known as mycolactones. These toxins possess cytotoxic and immunosuppressive activities and injection of purified mycolactone into animal models replicates the pathology seen in human BUs [7], [8]. Mycolactones are produced by large polyketide synthases encoded on the pMUM series of megaplasmids in members of the *M. ulcerans/Mycobacterium marinum* complex known as mycolactone producing mycobacteria (MPM) [9], [10]. Three polyketide synthase genes (*mlsA1*, *mlsA2* and *mlsB*) encode the mycolactone synthesis machinery, whilst a number of accessory genes that appear essential for mycolactone production are also present on the pMUM plasmids [9]. Furthermore, these plasmids contain a number of predicted protein-coding DNA sequences (CDS), with products of unknown function, the majority of which are conserved amongst different strains of MPM [11]. Several studies indicate that all the MPM (including *M. ulcerans*) diverged from a common *M. marinum* progenitor, through acquisition of the pMUM plasmid [10], [12], [13]. *M. marinum* strains share greater than 98% DNA identity with *M. ulcerans* [10], [12], [13] but they do not contain pMUM and do not make mycolactone, although some MPM that were given species names prior to the discovery of pMUM plasmids are confusingly referred to as *M. marinum, M. “liflandii”* and *M. pseudoshottsii* [14], [15], [16]. For simplicity, here all mycolactone producing mycobacteria are referred to as *M. ulcerans* and the closely related, non-mycolactone producing strains are referred to as *M. marinum*, in accordance with recent recommendations [17].

There are several laboratory-based methods for the diagnosis of BU in humans. These strategies currently include the direct examination of Ziehl-Neelsen stained smears, culture of *M. ulcerans* and PCR from swabs or tissue samples. Microscopy based on the Ziehl-Neelsen stain from BU swabs or biopsies is quick, however, several studies have shown that the sensitivity of this method is highly variable (40 – 80%, depending on the laboratory) [18], [19]. Culture of *M. ulcerans* from a suspect lesion remains the gold standard for diagnosis, however, due to the long incubation times required (up to 12 weeks) and low sensitivity it is not appropriate for pre-treatment diagnosis [20]. PCR for the *M. ulcerans* specific insertion sequence IS*2404* was first validated as a diagnostic test in 1997 [21] and several studies have shown it to be the most sensitive of the currently employed diagnostic techniques [20], [22], [23]. However, high reagent costs and the need for specialized equipment and trained staff to perform and interpret PCR restricts its use to larger, central laboratories. Thus none of these approaches are suited for use in rural African regions where BU is endemic and the World Health Organization has designated the development of new approaches to the diagnosis of *M. ulcerans* infection a research priority.

Early attempts to develop diagnostics for BU relied upon injection of Burulin (a crude *M. ulcerans* whole cell lysate) into individuals and waiting for the development of a delayed-type hypersensitivity response, akin to the Mantoux test [24]. Whilst those in the active stage of disease had strong reactions to Burulin, the majority also reacted to PPD, suggesting that there was significant cross-reactivity amongst mycobacterial antigens. Subsequently it was shown that BU patients develop serum antibodies to whole cell lysate of *M. ulcerans* [25], as well as culture filtrate proteins [26], [27]. However, these studies also found that up to 37% of control subjects had positive antibody responses to *M. ulcerans* proteins, again indicating that a significant degree of cross reactivity with other mycobacteria exists [25], [26], [27].

We reasoned that the identification of specific *M. ulcerans* antigens may help to overcome some of the difficulties associated with cross-reactivity to conserved mycobacterial antigens and accelerate efforts to develop a rapid diagnostic test. A recent increase in the number of mycobacterial genome sequences available has provided an opportunity to comprehensively identify proteins that are unique to *M. ulcerans*. We identified 45 *M. ulcerans* candidate antigens and then used PCR to assess their distribution in 26 *M. ulcerans* and 30 *M. marinum* strains. We then determined the reactivity of these proteins to sera from BU patients by ELISA. Here we show that a subset of these proteins is conserved amongst all *M. ulcerans* strains and that some of these proteins may be useful in seroepidemiological studies of Buruli ulcer.

## Methods

### Ethics statement

Ethics approval for serum collection was obtained from the National Ethics Committee of the Ministry of Health in Benin and the Human Ethics Committee of the Faculté des Sciences de la Santé, University of Abomey-Calavi, Benin. Informed consent was obtained for every participant. For children involved in this study, written, informed consent was obtained from their parents or legal guardians.

### Bacterial strains

Cloning of *M. ulcerans* unique sequences was performed in *E. coli* TOP10 cells (Invitrogen). The mycobacterial strains used in this study and their characteristics are listed in Table S1.

### Identification of *M. ulcerans* unique genes

Sequences unique to *M. ulcerans* were identified first by bioinformatic comparisons between the *M. ulcerans* genome and other recently completed mycobacterial genomes, using BLASTCLUST as described [28]. The completed and unfinished genomes utilized in this study were (Name, Genbank accession number): *M. abcessus* CIP104536 (NC_010397), *M. avium* subsp. p*aratuberculosis* K10 (NC_002944), *M. bovis* BCG str. Pasteur 1173P2 (NC_008769), *M. gilvum* PYR-GCK (NC_009338), *M. intracellulare* ATCC 13950 (NZ_ABIN00000000), *M. kansasii* ATCC 12748 (NC_ACBV00000000), *M. leprae* TN (NC_002677), *M. marinum* M (NC_010612), *M. smegmatis* mc^2^ 155 (NC_008596), *Mycobacterium sp.* KMS (NC_008705), *Mycobacterium sp.* JLS (NC_009077), *Mycobacterium sp.* MCS (NC_008146), *M. tuberculosis* C (NZ_AAKR00000000), *M. tuberculosis* CDC 1551 (NC_002755), *M. tuberculosis* F11 (NC_009565), *M. tuberculosis* H37Rv (NC_000962), *M. tuberculosis* H37Ra (NC_009525), *M. tuberculosis* Haarlem (NZ_AASN00000000), *M. avium* 104 (GenBank accession NC_008595), *M. ulcerans* Agy99 (NC_008611), and *M. vanbaalenii* PYR-1 (NC_008726). Forty-seven chromosomally encoded sequences and 54 plasmid-encoded sequences were initially selected as being *M. ulcerans* specific with no ortholog present in any other mycobacterial genome. Orthologues were defined as predicted amino acid sequences with greater than 85% amino acid identity to *M. ulcerans* proteins. Further criteria were applied to restrict the number of coding sequences (CDS) to investigate (see results section). Due to the high level of genetic relatedness between *M. ulcerans* and *M. marinum* strains, *M. marinum* and *M. ulcerans* strains spanning the known genetic diversity were then tested for these CDS by PCR using standard conditions and the addition of 5% dimethyl sulfoxide.

### DNA cloning and sequencing

Primers were designed to selected CDS to include a 5′ CACC sequence for cloning into the Gateway entry vector pENTR/SD/D-TOPO (Invitrogen) (see Table S2 for a list of oligonucleotides used in this study). CDS were PCR amplified using either *Pfu* Turbo (Stratagene) or KOD (Novagen) DNA polymerases under standard reaction conditions. Gateway cloning reactions were performed according to the manufacturer's instructions into the entry vector pENTR/SD/D-TOPO and positive clones were confirmed to be in-frame by DNA sequencing. Subcloning of the sequences of interest into one of three different *E. coli* expression vectors, pDEST17, pET-DEST42 or pBAD-DEST49 (Invitrogen), was accomplished using LR clonase enzyme (Invitrogen) according to manufacturer's instructions.

### Protein expression

For pDEST17 or pET-DEST42 constructs, overnight cultures of *E. coli* Rosetta 2 (Novagen), Rosetta-gami (Novagen), or C43 [29] containing the cloned expression constructs were grown and used to inoculate 200 ml volumes of Luria-Bertani broth containing 100 µg/ml ampicillin and 30 µg/ml chloramphenicol. Cells were grown at 37°C with shaking until they reached an optical density at 600 nm of approximately 0.8, at which point they were induced with IPTG (Isopropyl β-D-1-thiogalactopyranoside) at a final concentration of 1 mM. Cultures were incubated for a further 4 hrs at 37°C with shaking. Cells were then harvested by centrifugation.

For expression of proteins from pBAD-DEST49, *E. coli* DH10B cultures containing the appropriate constructs were grown as described above (without the addition of chloramphenicol) and were induced with a final concentration of 0.2% L-arabinose.

### Polyhistidine-tagged protein purification

Recombinant *M. ulcerans* fusion proteins that displayed high levels of expression from either of the three expression vectors were purified under denaturing conditions using Talon metal affinity resin (Clontech). Briefly, cell pellets from 200 ml cultures post-induction were resuspended in 10 ml wash buffer (300 mM NaCl, 50 mM NaH_2_PO_4_, 8M urea, pH 7.0). Samples were sonicated on ice for 3×30s and the sonicate was then pelleted for 20 min at 12,500× *g*. The clarified lysate was added to 1 ml prepared resin and incubated at room temperature for 30 min. The solution was drained from the column and the column was washed with 5 ml of wash buffer for 10 min at room temperature with gentle agitation. This was repeated twice, after which the protein was eluted from the column in wash buffer containing 150 mM imidazole, followed by a final elution using wash buffer containing 200 mM imidazole. Eluates were analyzed by SDS-PAGE and dialyzed against wash buffer to remove imidazole.

### Sera collection

Serum samples were collected from 93 individuals, comprising three groups. Group 1 (Patient group) consisted of 30 BU patients living in villages near the Ouémé river in the Ouémé province of Benin, where Buruli ulcer is highly prevalent (8–20 cases per 1000 individuals [30]). The patients (11 female and 19 male, aged 3–74 years) were recruited from the Centre de Diagnostic et de Traitement de l'Ulcère de Buruli in Pobè, Benin. Diagnosis of Buruli ulcer was made by Ziehl-Neelsen staining of material taken from swabs of the lesions or directly from biopsy material and subsequently confirmed by IS*2404* PCR. Group 2 (Endemic Controls) consisted 24 participants (14 female and 10 male aged 5–72 years) with no history of BU but from the same villages in the Ouémé province as the patients. Group 3 (Non-endemic Controls) consisted of 30 participants (15 female and 15 male), living in villages around Ouidah (Atlantic province), a non-endemic coastal area for BU in Benin, approximately 150 km southwest of Pobè [30]. BCG vaccination exposure (based on the presence of a scar) for all three groups was approximately 60%. Serum was prepared from 8 ml of blood for each participant and aliquots of each sample were stored at −80°C.

### SDS-PAGE and Western blotting

For analysis of purified protein reactivity by western blotting, purified proteins were separated by SDS-polyacrylamide gel electrophoresis (PAGE) on 12% polyacrylamide gels under reducing conditions, as described previously [31]. The gel was then transferred to nitrocellulose membranes in Tris-glycine buffer containing 20% methanol. Membranes were blocked in 5% skim milk, PBS with 0.1% Tween-20. Membranes were then incubated with human sera at 1/500 dilution. Prior to incubation with anti-human IgG horseradish-peroxidase (HRP) conjugated secondary antibody, the membranes were washed repeatedly in PBS-0.1% Tween-20. Bands were detected by chemiluminescence using the Western lightning kit (Perkin Elmer).

### Detection of human antibodies against potential *M. ulcerans* antigens by ELISA

Purified recombinant *M. ulcerans* proteins were adsorbed to polystyrene 96-well plates (Costar, Corning) at 0.5 µg per well in carbonate coating buffer (pH 9.6) and incubated at 4°C overnight. Plates were washed five times with PBS (pH 7.2) containing 0.05% Tween 20 (Sigma-Aldrich) (PBS-T) and then five times with PBS before being blocked with 100 µl 5% fetal bovine serum in PBS. Plates were washed as described above and 50 µl human sera (diluted 1/100 in PBS with 0.5% BSA) was added to each well. Plates were incubated for 2 hrs at room temperature before being washed as described above. 50 µl of goat anti-human IgG-HRP (Millipore) diluted at 1/2500 in PBS with 0.5% BSA was added to each well and incubated at room temperature for 1 hr, followed by washing as described above. Plates were developed by the addition of 50ul of ABTS solution (Invitrogen) to each well, incubated for 5 minutes and the reaction was stopped by the addition of 50 µl of 0.01% sodium azide, 0.1M citric acid. The colour change reaction was measured at 405 nm in Multiskan Ascent microplate reader (Thermo Fisher Scientific). Positive control wells were incubated with anti-6xHis-HRP antibody (Roche Applied Science).

### Data analysis

ELISA test results were analysed using GraphPad Prism version 5.0c (GraphPad Software, San Diego California USA). One-way ANOVA with Bonnferroni's post-test was used to compare OD values for patient, endemic controls and non-endemic control groups whose distributions approximated normality. ELISA data with non-gaussian distributions were analysed by the Kruskal-Wallis test with Dunn's post test. Cut-off scores for determination of inclusion of proteins for analysis were determined as the mean OD of the control population. Receiver-operator characteristic (ROC) analysis was used to determine the best cut-off value and associated sensitivity and specificity that discriminated individuals living in BU endemic areas from those living in non-endemic areas.

## Results

### Identification of potential *M. ulcerans* antigens

Forty-seven chromosomally-encoded CDS, potentially unique to *M. ulcerans* were identified by bioinformatic comparison of the *M. ulcerans* genome to 21 other mycobacterial genomes (see Materials and Methods for genomes used). We then excluded CDS <50 codons in length and the insertion sequence elements IS*2404* and IS*2606* and also applied the following criteria to include only those CDS likely to generate an immune response: i) predicted membrane association; ii) predicted secretion signal; or iii) previously confirmed as expressed in a proteomic study [32], resulting in 34 chromosomal CDS of interest. We also identified 33 unique CDS on the pMUM001 plasmid and in addition we included in our analysis the sequences encoding the 12 unique functional domains of the mycolactone polyketide synthases (MlsA1, MlsA2 & MlsB) as representative of the entire genetic variability present in this locus [9].

Due to the close genetic relationship between *M. ulcerans* and *M. marnium* and the knowledge that *M. marinum* M, does not reflect the genetic diversity in the *M. marinum* complex [10], 30 genetically diverse *M. marinum* isolates were tested for the presence of the selected chromosomal CDS by PCR. The *M. marinum* strains do not contain pMUM plasmids, so these isolates were not tested for the presence of any of the plasmid CDS. Eleven of the CDS identified as *M. ulcerans* specific by bioinformatic comparisons were found to be present in at least one of the other *M. marinum* strains tested and were thus excluded from further analyses (Table 1).

**Table 1:** Percentage of *M. marinum* and *M. ulcerans* strains containing selected *M. ulcerans* Agy99 sequences.

CDS	% MM^b^ strains	% MU^c^ strains	% MU^c^ African strains	CDS	% MU^c^ strains	% MU^c^ African strains
MUL_0027	3.7	-	-	MUP002	100	100
MUL_0076	51.8	-	-	MUP003	100	100
MUL_0503	92.6	-	-	MUP004	100	100
MUL_0508	0	88.5	100	MUP006	80.8	100
MUL_0510	0	88.5	100	MUP007	92.3	100
MUL_0511	0	92.3	100	MUP013	92.3	100
MUL_0512	0	84.6	100	MUP014	88.5	100
MUL_0513	0	84.6	100	MUP015	96.2	100
MUL_0515	0	76.9	100	MUP016	80.8	100
MUL_0516	0	88.5	100	MUP017	61.5	100
MUL_0517	0	96.2	100	MUP018	80.8	100
MUL_0526	29.6	-	-	MUP019	73.1	100
MUL_0527	48.1	-	-	MUP020	80.8	100
MUL_0551	0	73.1	100	MUP021	88.5	100
MUL_0552	0	88.5	100	MUP023	100	100
MUL_0998	0	92.3	100	MUP024	100	100
MUL_0999	0	92.3	100	MUP038	92.3	90
MUL_1001	0	92.3	100	MUP045	100	100
MUL_1135	7.5	-	-	MUP046	96.2	100
MUL_2590	44.4	-	-	MUP057	46.2	100
MUL_2831	0	100	100	MUP064	46.2	100
MUL_2832	0	96.2	100	MUP065	53.8	100
MUL_3210	74.1	-	-	MUP066	88.5	100
MUL_3212	0	100	100	MUP067	73.1	100
MUL_3214	0	96.2	100	MUP068	65.4	100
MUL_3215	0	92.3	100	MUP070	65.4	100
MUL_3216	0	96.2	100	MUP071	88.5	100
MUL_3217	0	100	100	MUP074	88.5	100
MUL_3218	0	69.2	100	MUP075	88.5	100
MUL_3230	0	100	100	MUP076	84.6	100
MUL_3440	66.6	-	-	MUP078	92.3	100
MUL_3828	0	69.2	100	MUP079	92.3	100
MUL_4213	44.4	-	-	MUP080	96.2	100
MUL_4217	44.4	-	-	PKS domains^a^	100	100

a = All sequences encoding the 12 PKS domains are found in 100% of mycolactone producing mycobacteria.

b = *M. marinum*;

c = *M. ulcerans*.

The pMUM plasmids are known to vary in both size and gene content between *M. ulcerans* strains [11], [33], and so we tested a selection of 26 geographically distinct *M. ulcerans* isolates, by PCR, for the presence of each of the plasmid sequences (Table 1). Additionally, we examined these strains for the presence of each of the selected chromosomal sequences by PCR, and found that six of the plasmid CDS and four of the chromosomally encoded CDS were present in all of the *M. ulcerans* strains tested (Table 1). Because the focus of our study is to develop diagnostic reagents for use in African patients where the need for BU control is most urgent, we particularly focused on the distribution of sequences of interest in African isolates of *M. ulcerans.* The 23 *M. ulcerans* specific chromosomal CDS were found in all 10 of the African *M. ulcerans* strains tested. As expected, the plasmid-borne mycolactone polyketide synthase (*mls*) sequences were found in all *M. ulcerans* strains tested, and also 32 of 33 non-*mls* plasmid sequences were present in all African strains. MUP038 was absent by PCR from a single African strain (*M. ulcerans* strain Kob), which confirms previous findings for this strain showing it has a plasmid DNA deletion [33] (Table 1).

The completed list of *M. ulcerans* unique CDS selected for further study included 13 chromosomal and 30 plasmid CDS (12 *mls* and 18 non-*mls*) and is shown in Table S3. In addition we included as positive controls two CDS encoding proteins Hsp65 and Hsp18 (MUL_2232) which have been reported to be antigenic but which are present in other mycobacteria species [34], [35]. This resulted in a total of 45 proteins under investigation.

### Cloning, expression and purification of *M. ulcerans* sequences in *E. coli*

All 45 of these selected *M. ulcerans* sequences were amplified from strain Agy99 genomic DNA and 44 were cloned using the Gateway system into at least one of three different expression vectors, pDEST17 (N-terminal 6xHis tag), pET-DEST42 (C-terminal 6xHis tag) or pBAD-DEST49 (N-terminal thioredoxin tag, C-terminal 6xHis tag). We then successfully expressed 37 of 44 (84.1%) of the target sequences in *E. coli* as either N-terminal or C-terminal 6xHis tagged proteins or both at or near their predicted molecular weight (Table S3). Whilst 37 proteins could be expressed in quantities sufficient for western blotting, only 33 of these 37 were able to be produced in quantities sufficient for ELISA analysis.

Seven proteins were unable to be expressed in *E. coli* as either N- or C-terminal 6xHis tagged fusions. Expression of two of these proteins (MUP016 and MUP017) from either pDEST17 or pET-DEST42 proved to be toxic to *E. coli*. Sequencing of the remaining five constructs showed in-frame fusions had been made and western blots on WCLs of *E. coli* bearing these constructs showed an absence of recombinant protein expression after induction (data not shown). Further attempts were made to salvage these seven proteins by cloning into pBAD-DEST49, a vector with arabinose inducible expression, an N-terminal thioredoxin tag and a C-terminal 6xHis tag, designed to improve protein expression and solubility, and by using *E. coli* strain C43, which has been optimized for the expression of toxic or membrane proteins [29], [36]. However, these efforts were also unsuccessful and so these proteins were unable to be produced to levels sufficient for purification.

### Diagnostic potential of *M. ulcerans* specific antigens

To evaluate the potential for these proteins to be used to diagnose *M. ulcerans* infection or assess exposure to the bacterium, sera was collected from 39 IS*2404* PCR confirmed BU patients (designated: patient) and 24 controls with no past or current diagnosis of BU (designated: endemic control). Both these groups came from the high BU prevalence Ouémé region in Benin. Sera were also obtained from 30 additional control subjects who lived in the BU non-endemic Ouidah region of Benin (designated: control). A pre-study decision was taken to analyze in these three groups rather than only patients versus non-endemic controls as previous studies had indicated the possibility of asymptomatic exposure to *M. ulcerans* in BU endemic areas [34].

**Figure 1:**
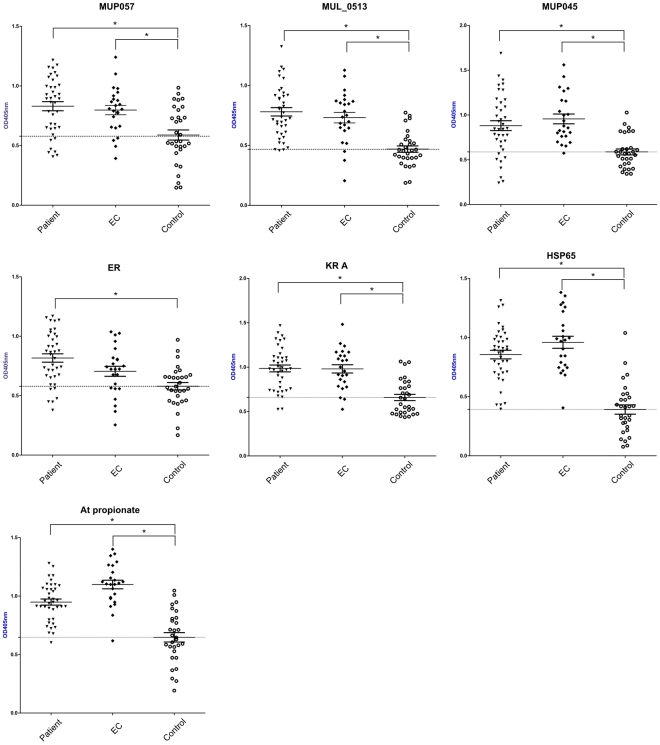
ELISA results for seven *M. ulcerans* proteins capable of discriminating between patients and controls. Comparison of patient, endemic control (EC) and non-endemic control sera reactivity to seven *M. ulcerans* antigens that showed an ability to discriminate between patient and control sera. Mean OD405nm readings for each group and standard error of the mean are shown. The horizontal dotted line in each figure represents the mean OD405nm reading of the control group. * = *p*<0.05.

Screening of 33 proteins by ELISA uncovered significantly higher IgG antibody responses in patients compared to non-endemic controls for the following seven proteins; MUP045, MUP057, MUL_0513, Hsp65, and the mycolactone Mls domains (AT-propionate, ER, and KR-A domains) (*p*<0.05, Figure 1). However, IgG responses of the endemic controls were not significantly different to those of patients for any of the seven proteins (Figure 1). Endemic controls had IgG responses that were significantly higher than those of non-endemic controls for the six of these seven proteins, suggesting that residents of endemic regions with no history of BU might have been exposed to *M. ulcerans*. The remaining 26 proteins, including the positive control antigen MUL_2232 (Hsp18), had no ability to elicit discriminatory antibody responses between any groups (Figure S1).

**Figure 2:**
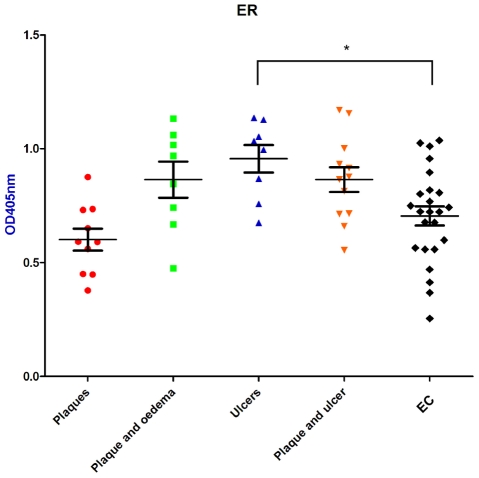
Analysis of BU patient serum reactivity by disease state. Patient antibody responses were analysed by disease state for the mycolactone polyketide synthase ER domain. Means and SEM of each group are shown. * = *p*<0.05.

When serum antibody responses for the proteins shown in Figure 1 were analysed according to disease state (*i.e,* plaque, plaque and oedema, ulcer, or plaque and ulcer), there was no significant difference in reactivity between disease state and endemic controls for each protein except for ER (Figure 2). Mean ELISA absorbance values for sera from patients with ulcers were significantly higher in their reactivity to the ER domain of the mycolactone PKS than endemic controls and other lesion types (*p*<0.05, Figure 2).

### Seroepidemiological potential of *M. ulcerans* specific antigens

**Figure 3:**
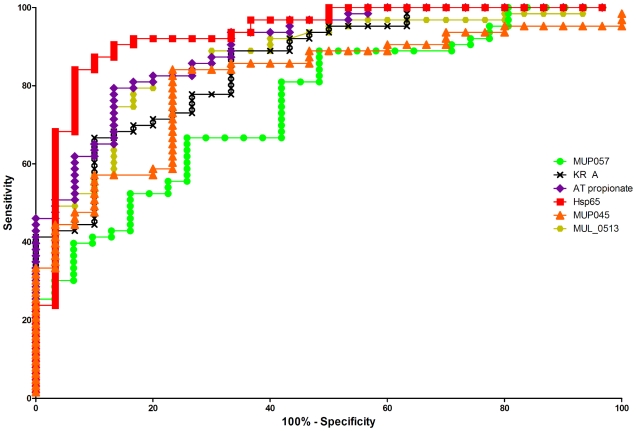
Receiver operator characteristic (ROC) analysis of six *M. ulcerans* specific proteins. ROC curves are shown for the six *M. ulcerans* specific proteins detailed in Figure 1 (MUP045, MUP057, MUL_0513, Hsp65, AT propionate and KR A).

We examined six of the seven proteins shown in Figure 1 to test if they could effectively distinguish subjects from BU endemic and BU non-endemic regions of Benin. We performed receiver-operator curve (ROC) analyses for each of these six antigens by combining the ELISA absorbance values for patient and endemic control groups (collectively referred to as BU-endemic) (Table 2) to calculate sensitivity and the non-endemic control group for specificity. ROC curves for each of the six proteins are shown in Figure 3. The ELISA OD cutoffs that maximized the accuracy of each of the six antigens are shown in Table 2 and the results indicated that a number of these proteins might be useful in determining new *M. ulcerans* endemic areas. In particular MUL_0513 and two of the Mls domains (AT-propionate, KR-B) all produced high area under curve (AUC, >0.8), and good sensitivity (>70%) and specificity (>80%). However the best antigen was Hsp65, with AUC of 0.932, 84.1% sensitivity and 93.3% specificity at an OD cut-off of 0.693. Hsp65 also had the highest likelihood ratio (12.6), indicating that an individual living in a BU endemic area is 12.6 times more likely to have a positive Hsp65 test than an individual residing in a non-endemic area.

**Table 2:** Receiver-operator characteristics for proteins with predictive value for living in a BU endemic area.

Antigen	AUC^a^	95% CI	Cut-off	Sensitivity (%)	Specificity (%)	Likelihood ratio^b^	NPV^c^
MUP045	0.810	0.720–0.901	0.824	57.1	90.0	5.7	0.61
MUP057	0.753	0.652–0.854	0.893	39.7	93.5	6.2	0.57
MUL_0513	0.873	0.799–0.946	0.622	74.6	86.7	5.6	0.70
Hsp65	0.932	0.873–0.991	0.693	84.1	93.3	12.6	0.77
AT propionate	0.897	0.832–0.962	0.896	79.4	86.7	6.0	0.70
KR A	0.856	0.777–0.934	0.850	69.8	83.3	4.2	0.68

a AUC, area under curve;

b Likelihood ratio represents the likelihood that someone with a positive test will live in a BU endemic area, *i.e*. a likelihood ratio of 5.0 means someone with a positive test is 5 times more likely live in BU endemic area than an individual with a negative test;

c NPV, negative predictive value.

## Discussion

The current diagnostic delay experienced by Buruli ulcer patients contributes to morbidity and increased economic hardship [37]. A diagnostic test that is rapid, inexpensive, sensitive, specific and suitable for in-field use would facilitate the timely diagnosis of BU and is a World Health Organization (WHO) research priority. Current laboratory diagnostics including culture, PCR, AFB microscopy and histopathology all have various limitations. We have used comparative genomics to identify *M. ulcerans* specific sequences to explore the usefulness of the encoded proteins for use in the serodiagnosis of Buruli Ulcer in endemic areas of West Africa.

Whilst bioinformatic methods were initially used to identify *M. ulcerans* specific CDS, eleven of these “*M. ulcerans* specific” CDS were found in at least one of the *M. marinum* strains tested (Table 1). These CDS are distributed across the *M. ulcerans* genome, and were all situated within regions of DNA that are not present in the *M. marinum* M genome. It is interesting to note that some of these sequences appear restricted to certain *M. marinum* sequence types that are genetically closer to *M. ulcerans* than the sequenced *M. marinum* M strain. For example MUL_4213 was restricted to the ST3 and ST5 strains tested and MUL_0027 was restricted to the ST3 strains tested. Some of these sequences may be useful as genotyping tools to more clearly delineate the relationships between *M. ulcerans* and *M. marinum* strains. These findings are consistent with the known high genetic relatedness of *M. ulcerans* and *M. marinum* [10], [13], and underlies the need for experimental validation of bioinformatic results.

Only nine *M. ulcerans* specific sequences were conserved amongst all mycolactone producing mycobacterial strains tested. However, in agreement with previous findings that African *M. ulcerans* strains form a close genetic complex with relatively little variation at the whole genome level [38], [39], [40], all selected chromosomal genes were present in all African *M. ulcerans* isolates that were examined. As pMUM plasmids are known to vary in size and gene content between *M. ulcerans* strains [11], [33], it is not surprising that only six of 33 non-PKS CDS were completely conserved. However, plasmid CDS may be the most specific to *M. ulcerans* as they appear to be restricted to mycolactone producing mycobacteria.

During the course of this work the sequences of two other pMUM plasmids from MPM became available [11] and revealed a number of differences between these plasmids with regards to the potential antigens chosen for this study. These differences can explain the inability to detect some of the selected CDS in MPM strains. For example, MUP006 orthologs in pMUM002 and pMUM003 contain a different 5′ nucleotide sequence, explaining the absence of a PCR product in some MPM strains. Conversely, MUP068 and MUP070 orthologs in pMUM001 and pMUM002 contain different 3′ nucleotide sequences. Furthermore, MUP065 appears to be a pseudogene in pMUM001, as its ortholog in pMUM002 and pMUM003 are significantly longer, and MUP067 is deleted from pMUM002 and pMUM003. All of these examples serve to highlight the genetic variability in the non-PKS component of these pMUM plasmids.

In this study 44 *M. ulcerans* specific sequences were cloned with 37 (84%) of these successfully expressed in *E. coli*. Seven proteins were unable to be expressed in multiple *E. coli* strains, and the lack of overexpressed protein was confirmed by western blotting (data not shown). Similar studies that have attempted to identify novel CDS in *M. avium* subsp. *paratuberculosis* have also shown variable rates of production of 6 x His tagged proteins (between 25 – 69% of cloned CDS able to be expressed) [41], [42], indicating that this tag may reduce expression of certain proteins. Furthermore, all proteins in this study were found to be insoluble under the expression conditions used, and six of the seven proteins that were unable to be expressed were predicted to be integral membrane proteins, with overexpression of these proteins possibly leading to toxicity. Both of these problems have been previously described in other mycobacterial protein expression studies [43], [44], [45]. Clearly, further optimization of the expression of these proteins, including the use of alternate proteins tags or different host-based expression systems (including mycobacteria) may be necessary to obtain useable amounts of protein for future testing. Recently, a Gateway-based vector has been developed for the overexpression of proteins in *M. smegmatis* and has shown that proteins which were previously insoluble in *E. coli* could be expressed in soluble form in *M. smegmatis* [46]. Furthermore, *M. tuberculosis* proteins have been successfully produced in the methylotrophic yeast *Pichia pastoris* with enhanced patient serum reactivity compared with those produced in *E. coli* [47], [48]. The future use of either of these systems for the proteins that proved difficult to produce in this study may be warranted to investigate their antigenicity.

We were able to confirm that patients with BU had significantly greater IgG antibody responses to seven of 33 purified *M. ulcerans* proteins as determined by ELISA compared with a control group (Figure 1). The inability of the remaining 26 proteins to discriminate between patients and controls is likely due to either the inability of a particular protein to elicit an antibody response or through shared epitopes with proteins from other sources leading to cross-reactive antibody responses. However, none of the proteins were able to discriminate between patients and control subjects from the same BU-endemic area and are therefore unlikely to be useful as a diagnostic test for *M. ulcerans* infection. By combining measurements of antibody responses to six antigens from people living in a BU endemic area, we were able to discriminate between subjects resident in endemic areas of Benin and controls living in a non-endemic area. Although it remains a possibility that these data reflect cross-reactive antibody responses to other mycobacteria, we suggest that the most likely interpretation of these findings is that many residents of BU-endemic areas have been exposed to *M. ulcerans*.

Somewhat surprisingly, the most discriminatory antigen was Hsp65, which has 94% homology to the same protein from *M. tuberculosis*, whilst antigens with no orthologues in other mycobacteria had lower sensitivity and specificity. Hsp65 is known to be an immunodominant antigen in the antibody response to mycobacteria [35] and we anticipated that all groups would have had significant levels of exposure to other mycobacteria from either BCG vaccination, tuberculosis infection, and from exposure to other environmental mycobacteria. We therefore anticipated that Hsp65 would be a sensitive but non-specific antigen. There may be several explanations for our findings. Firstly, patients and endemic controls were both from a region of high BU prevalence and therefore may have been recently and/or frequently exposed to *M. ulcerans*, leading to continual boosting of antibody responses that does not occur in the non-endemic control group. The fact that *M. ulcerans* elicits responses dominated by antibodies to Hsp65 at all stages of the infection in mice supports this hypothesis (35). Alternatively, it is possible that our patient and control groups differed in their exposure to mycobacteria other than *M. ulcerans*, and therefore the results do not reflect *M. ulcerans* specific antibody responses.

The small heat shock protein, Hsp18, had previously been identified as an immunodominant antigen that may be useful for predicting exposure to *M. ulcerans* [34]. Results from the current study, however, showed that Hsp18 was not able to discriminate between individuals living in an endemic area and those who reside in a non-endemic area. The reasons for these differences are unclear, however, a possible explanation may be that here, antibody responses were quantified by ELISA, as opposed to qualitative western blot analysis in the study by Diaz *et al* [34].

Although the other antigens we identified as *M. ulcerans* specific by our comparative genomic approach did not appear to be as sensitive or specific as Hsp65 in predicting individuals living in a Buruli endemic area, they nevertheless did show that significant antibody responses were detectable, and three antigens (MUL_0513, AT-propionate, KR-B) showed significant ability to discriminate individuals living in a BU endemic versus non-endemic area. Their use in combination may show additive effects and, in conjunction with case-control studies, may permit the development of an assay for detecting individuals exposed to *M. ulcerans*. More research in this direction would greatly assist efforts to uncover environmental reservoirs and understand transmission pathways of the bacterium.

Although a number of *M. ulcerans* specific sequences were investigated in this study, there were still a large number that were initially ruled out based on predictions of their cellular location or function. It is possible that some of these potential antigens may be good candidates for further analysis. Based on our findings of high response rates among patient and endemic control groups it appears unlikely that serological approaches will be useful for diagnosing Buruli ulcer. Indeed, the development of serodiagnostics for other mycobacterial diseases, such as tuberculosis, has proved difficult and many of the currently available serodiagnostics for mycobacteria have limited usefulness in the clinic [49], [50].

An alternative to BU serodiagnosis may be possible via direct antigen detection approaches. The extent and distribution of antigens within *M. ulcerans* lesions or within a patients circulation has not been defined, however, there is the possibility that some of the unique proteins described in this present study may be able to be developed as the basis for antigen capture assays. A diagnostic test based on this methodology would overcome the problems of cross-reactivity seen with tests based upon antibody responses, and would be more appropriate for resource limited areas than tests based on T-cell responses. This project has helped define the antigenic repertoire of *M. ulcerans* and research is continuing in this vein to investigate the feasibility of other diagnostic modalities.

## Supporting Information

Figure S1 ELISA results for 26 *M. ulcerans* proteins that showed no significant difference in reactivity between patients, endemic controls or non-endemic controls. Reactivity of individual patient and endemic control (EC) samples are shown. Mean reactivity of non-endemic control sera group is represented by a dotted horizontal line across each graph. Mean OD405 nm readings for each group and standard error of the mean are also shown.
(0.15 MB PDF)

Table S1 List of bacterial strains used in this study.
(0.12 MB DOC)

Table S2 Oligonucleotides used in this study.
(0.08 MB DOC)

Table S3 *M. ulcerans* genes tested in this study.
(0.06 MB DOC)
