# Supplementary figures and images for: Serological Evaluation of Mycobacterium ulcerans Antigens Identified by Comparative Genomics
Source: PLoS Negl Trop Dis. 2010 Nov 2;4(11):e872. doi: 10.1371/journal.pntd.0000872 (PMC2970529; doi:10.1371/journal.pntd.0000872)

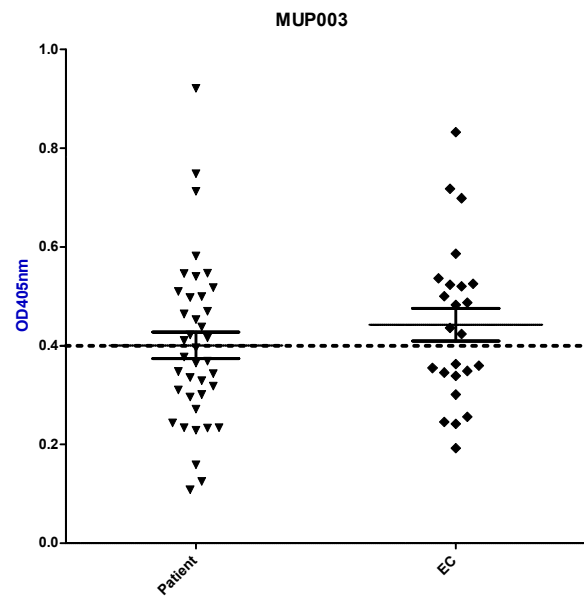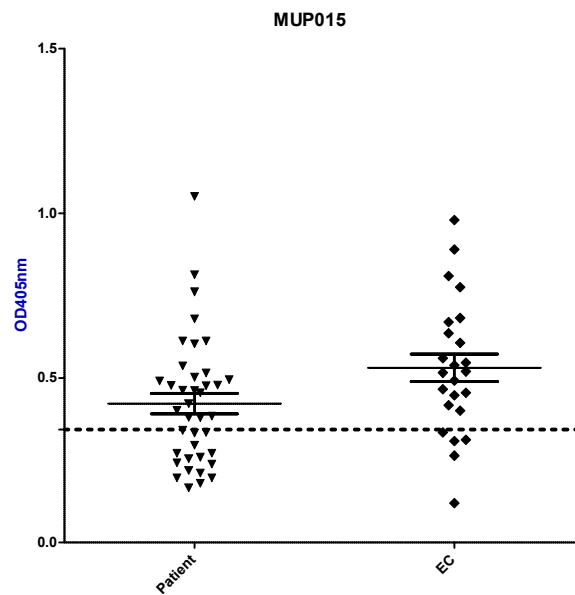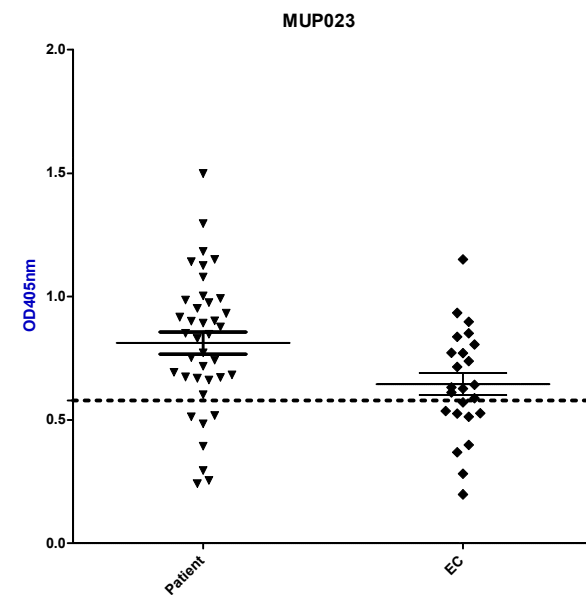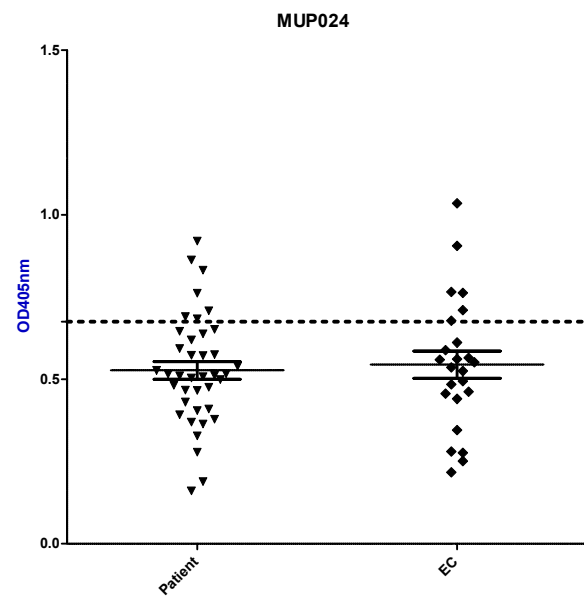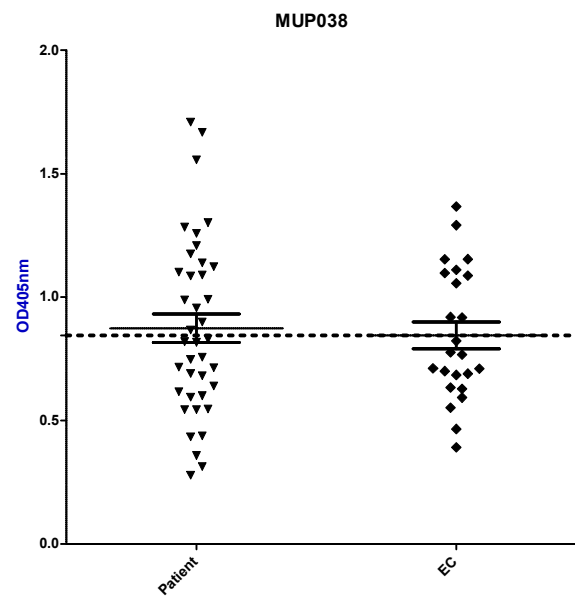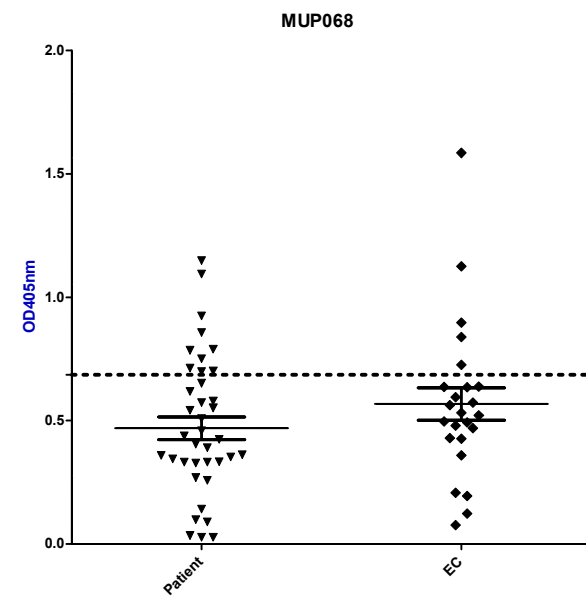

MUL\_0512

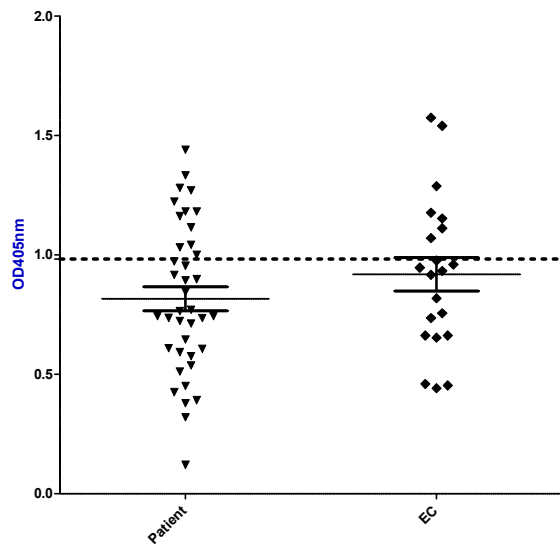

MUL\_0515

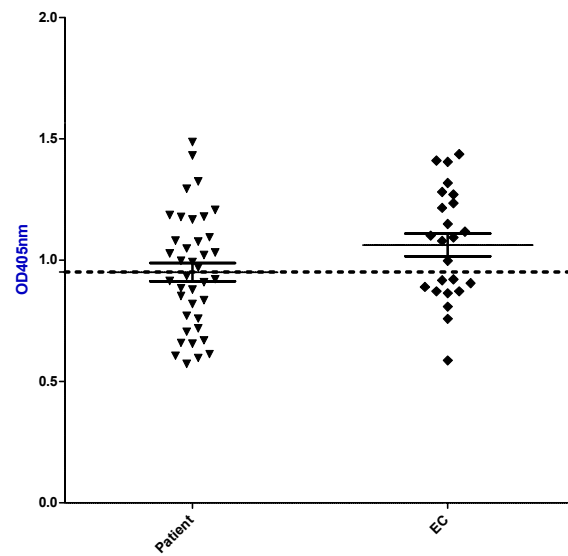

MUL\_0516

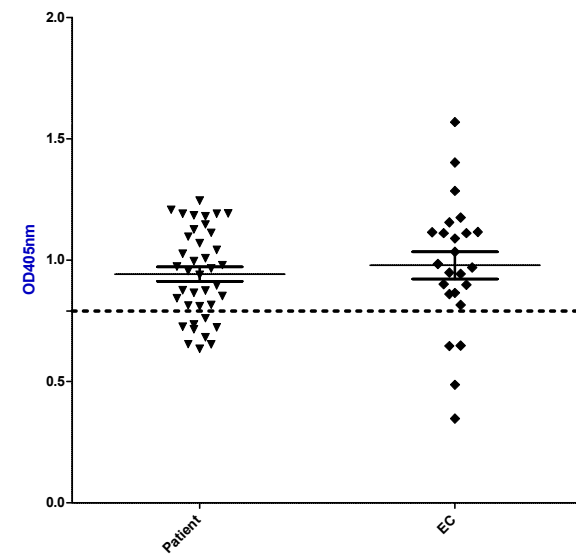

MUL\_0998

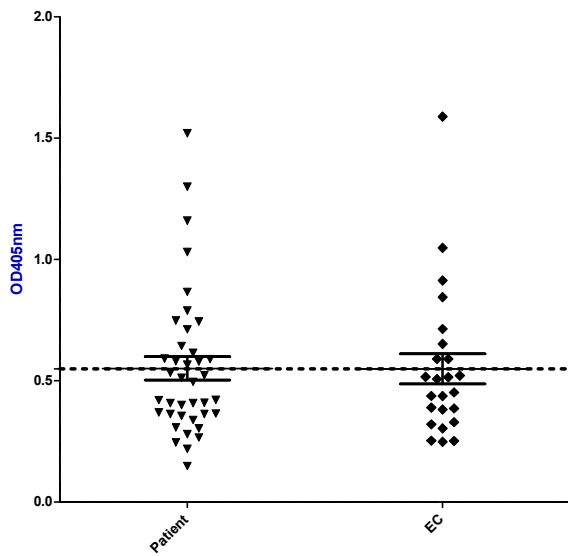

MUL\_1001

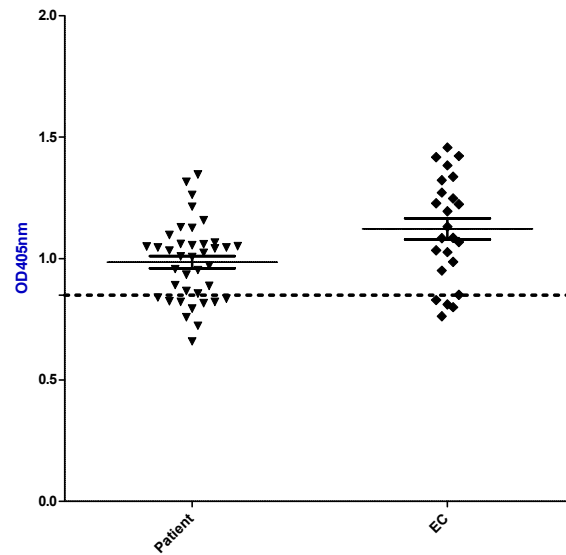

MUL\_2232

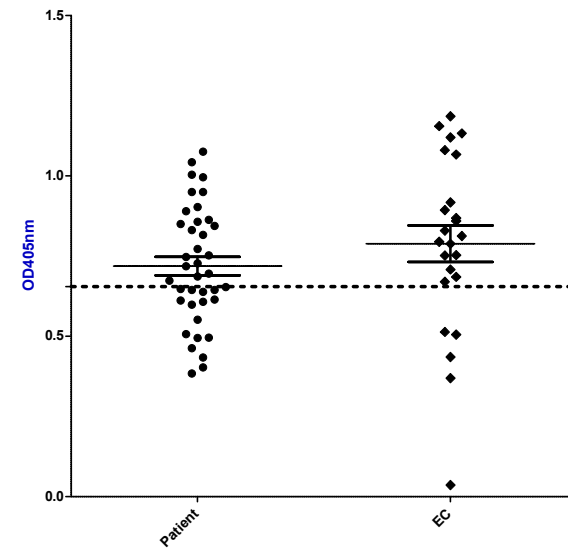

MUL\_2831

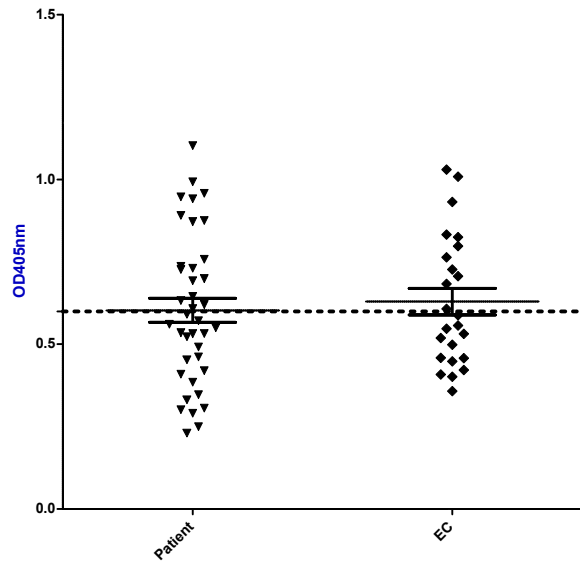

MUL\_3212

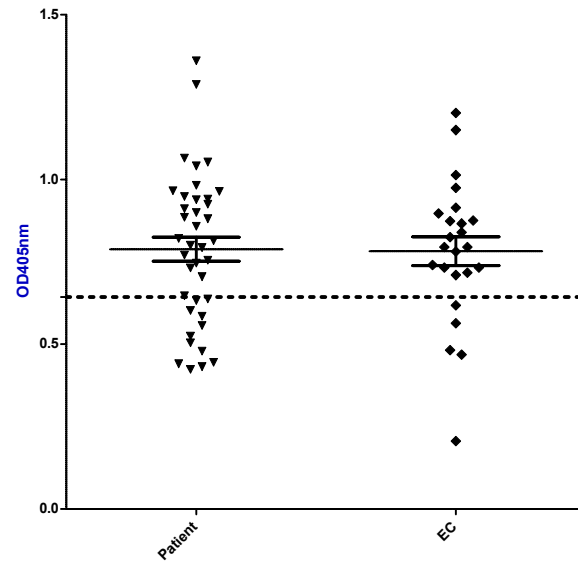

MUL\_3216

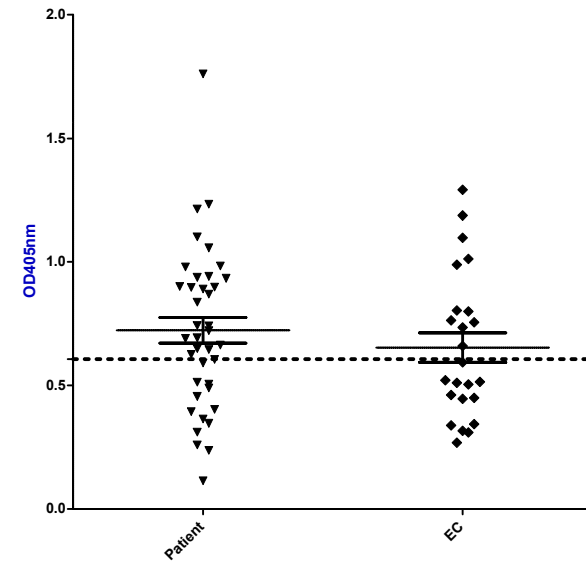

MUL\_3217

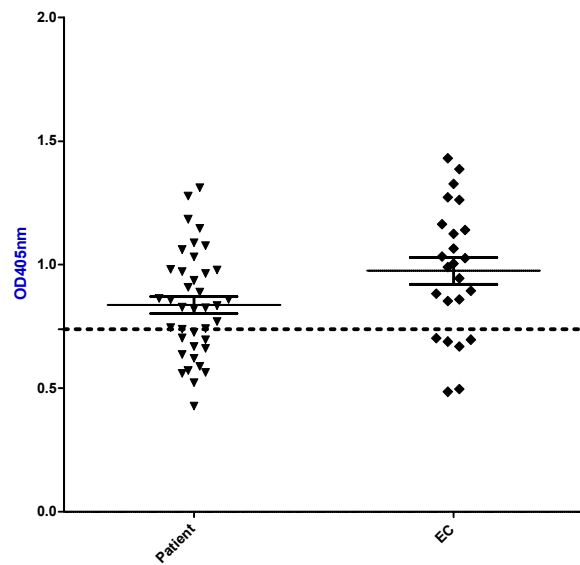

MUL\_3218

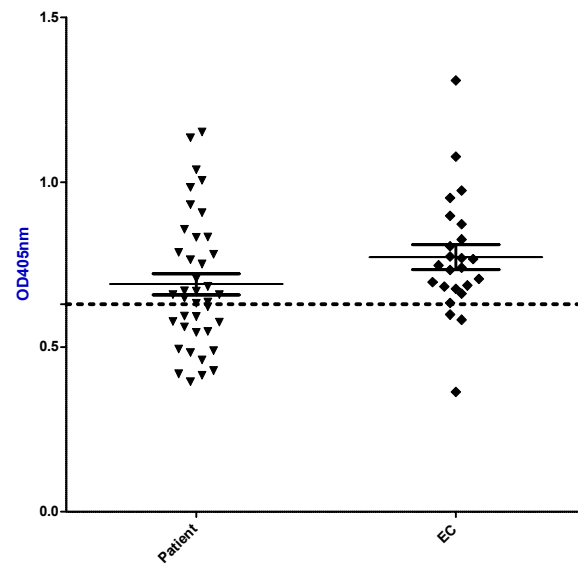

KS core

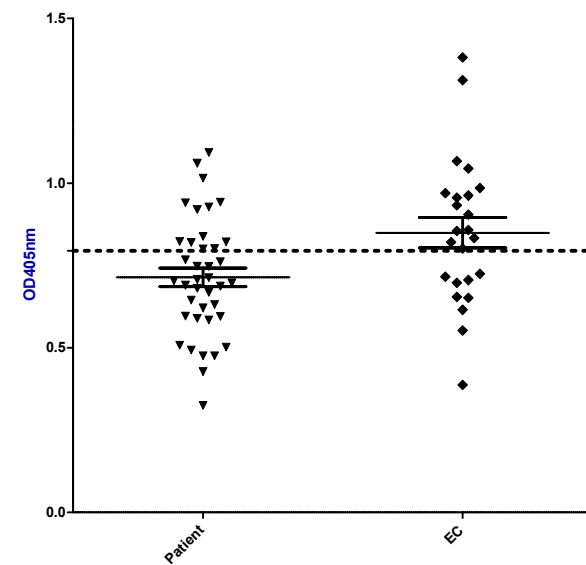

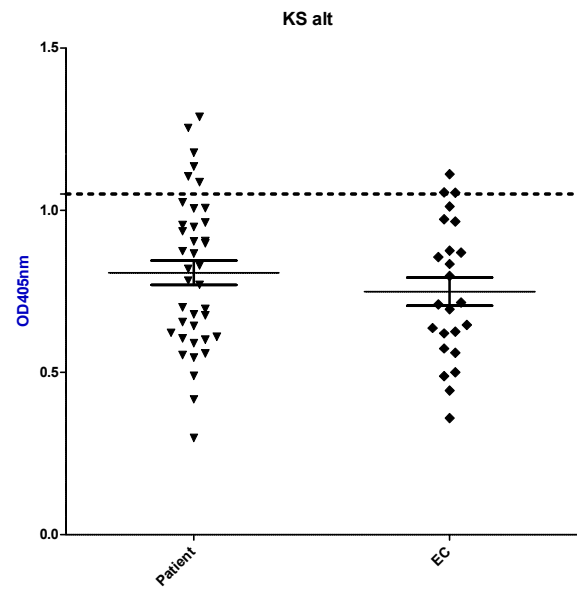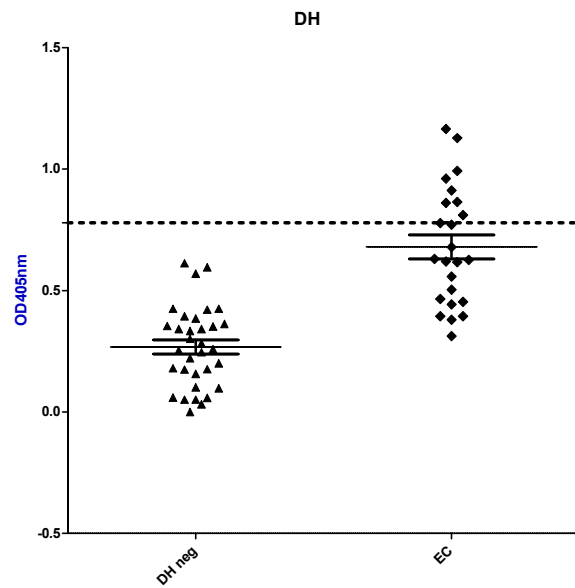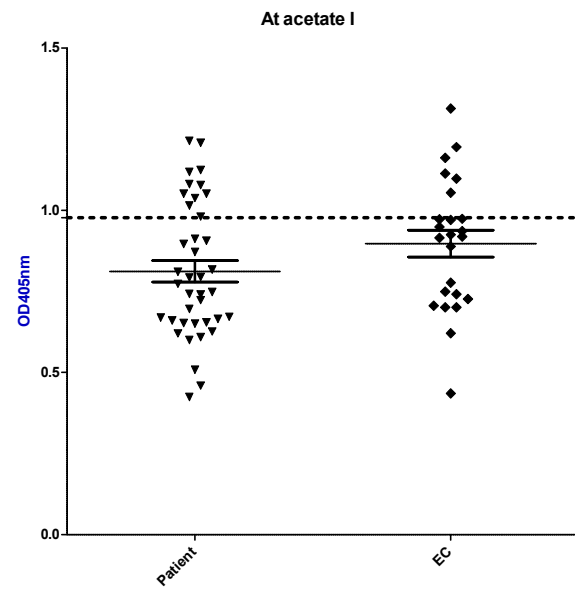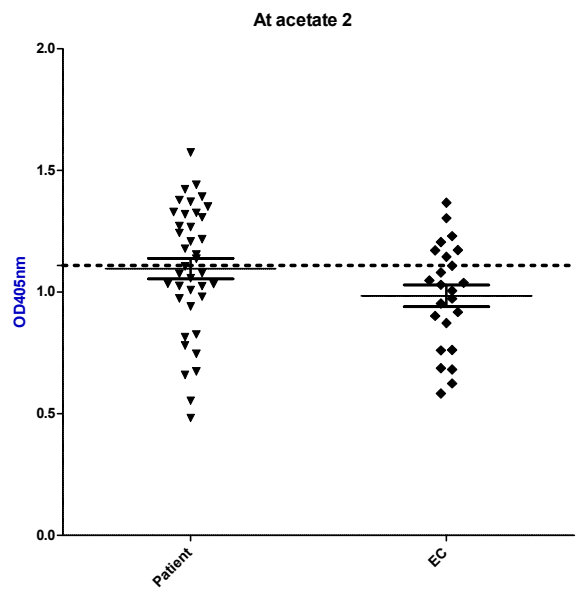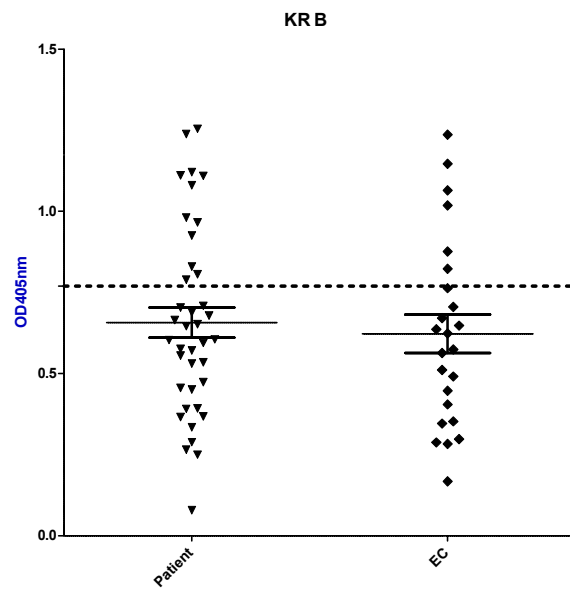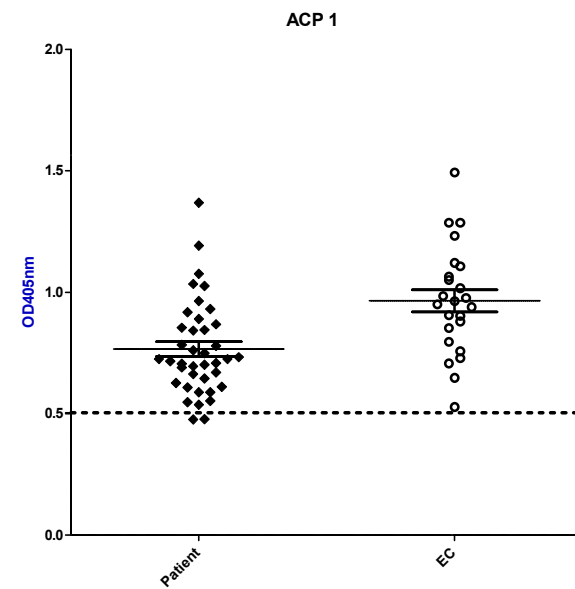

ACP 2

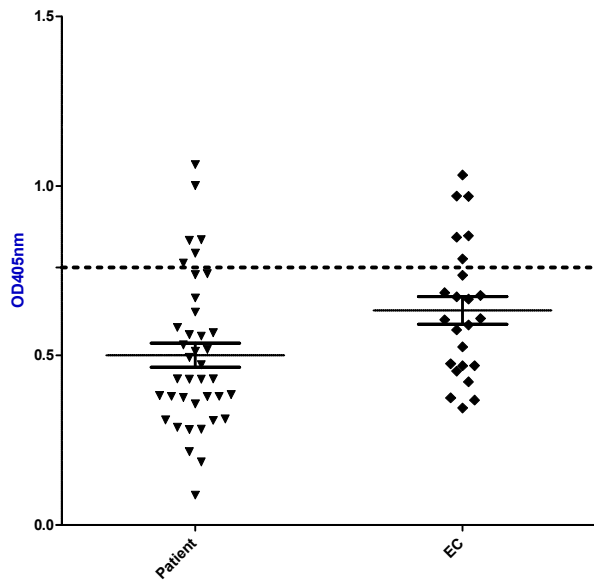

ACP 3

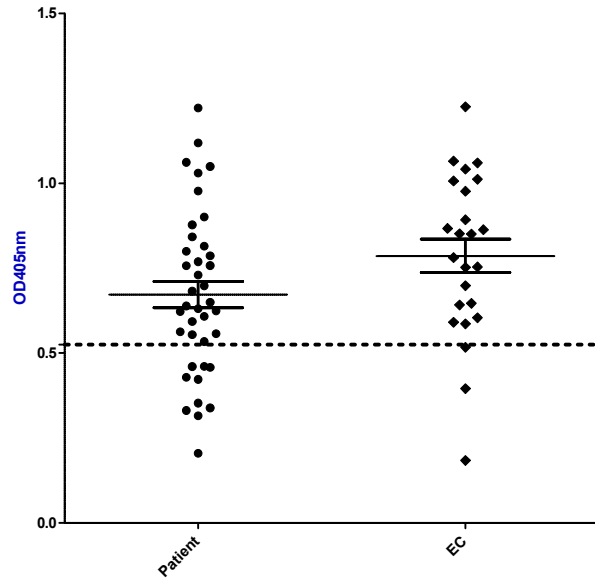

Supplement: Figure S1 — ELISA results for 26 M. ulcerans proteins that showed no significant difference in reactivity between patients, endemic controls or non-endemic controls. Reactivity of individual patient and endemic control (EC) samples are shown. Mean reactivity of non-endemic control sera group is represented by a dotted horizontal line across each graph. Mean OD405 nm readings for each group and standard error of the mean are also shown. (0.15 MB PDF) [file pntd.0000872.s001.pdf]
