# Supplementary material for: Serological Evaluation of Mycobacterium ulcerans Antigens Identified by Comparative Genomics
Source: PLoS Negl Trop Dis. 2010 Nov 2;4(11):e872. doi: 10.1371/journal.pntd.0000872 (PMC2970529; doi:10.1371/journal.pntd.0000872)
Supplement: Table S1 — List of bacterial strains used in this study. (0.12 MB DOC) [file pntd.0000872.s002.doc]

**Supplementary Table 1 – List of bacterial strains used in this study**

| Genus/Species | Strain name | Characteristics | Source^a^ | Reference |
| --- | --- | --- | --- | --- |
| *M. ulcerans* | Agy99 | Human clinical isolate, Ga district, Ghana |  | [1] |
|  | Kob | Human clinical isolate, Ivory Coast | IP |  |
|  | 5145 | Human clinical isolate, Benin | ITM | [2] |
|  | 9673 | Human clinical isolate, Zaire | ITM | [2] |
|  | 96-658 | Human clincal isolate, Angola | ITM |  |
|  | 94-856 | Human clinical isolate, Benin | ITM |  |
|  | ATCC 19423 | Human clinical isolate, Victoria, Australia | ATCC |  |
|  | 97-111 | Human clinical isolate, Benin | ITM |  |
|  | 5152 | Human clincal isolate, Congo | ITM |  |
|  | 97-610 | Human clinical isolate, Ghana | ITM |  |
|  | 97-680 | Human clinical isolate, Togo | ITM |  |
|  | JKD8045 | Human clinical isolate, Victoria, Australia | RCH | [2] |
|  | JKD8049 | Human clinical isolate, Victoria, Australia | VIDRL | This study |
|  | 13822/70 | Human clinical isolate, Queensland, Australia | QDRLMD | [2] |
|  | 94-1331 | Human clinical isolate, Papua New Guinea | ITM |  |
|  | ATCC 35840 | Human clinical isolate, Malaysia | ATCC |  |
|  | 94-1328 | Human clinical isolate, Malaysia | ITM | [3] |
|  | 98-912 | Human clinical isolate, China | ITM | [4] |
|  | 8765 | Human clinical isolate, Japan | ATCC | [5] |
|  | 5114 | Human clinical isolate, Mexico | ITM | [6] |
|  | 842 | Human clinical isolate, Surinam | ITM | [6] |
|  | L15^b^ | Isolated from diseased striped bass (*Morone saxitalis*), Chesepeake Bay, USA |  | [7] |
|  | 128FXT^b^ | Isolated from diseased frog, Berkeley, California, USA |  | [8] |
|  | CC240299^b^ | Isolate from diseased Koi (*Cyprinus carpio*), Israel |  | [9] |
|  | DL240490^b^ | Isolated from diseased sea bass (*Dicentrarchus labrax*), Red Sea, Israel |  | [9] |
|  | DL045^b^ | Isolated from diseased sea bass (*Dicentrarchus labrax*), Mediterranean sea, Greece |  | [9] |
| *M. marinum* | M | Human clinical isolate, California, USA | ATCC |  |
|  | 500525 | Human clinical isolate, Canberra, Australia | ICPMR |  |
|  | 993362605 | Human clinical isolate, New South Wales, Australia | ICPMR |  |
|  | 412214 | Human clinical isolate, New South Wales, Australia | ICPMR |  |
|  | JKD2394 | Human clinical isolate, Victoria, Australia | VIDRL |  |
|  | JKD2395 | Human clinical isolate, Victoria, Australia | VIDRL |  |
|  | MON6 | Human clinical isolate, Victoria, Australia | This study |  |
|  | JKD2396 | Human clinical isolate, Victoria, Australia | VIDRL |  |
|  | JKD2397 | Human clinical isolate, Victoria, Australia | VIDRL |  |
|  | ATCC 11565 | Human clinical isolate, Sweden | ATCC |  |
|  | MON10 | Human clinical isolate, Philadelphia, USA | RML | [3] |
|  | 471 | Human clinical isolate, Norway | RML |  |
|  | MON12 | Human clinical isolate, Victoria, Australia | This study |  |
|  | 472 | Human clinical isolate, Norway | RML |  |
|  | 991831797 | Human clinical isolate, New South Wales, Australia | ICPMR |  |
|  | 1542578 | Human clinical isolate, New South Wales, Australia | ICPMR |  |
|  | 992092077 | Human clinical isolate, New South Wales, Australia | ICPMR |  |
|  | 991961552 | Human clinical isolate, New South Wales, Australia | ICPMR |  |
|  | NCTC2275 | Saltwater fish, Philidelphia, USA | NCTC |  |
|  | MON19 | Fish isolate, Victoria, Australia | This study |  |
|  | MON20 | Fish isolate, Philadelphia, USA | ATCC |  |
|  | 99/84 | Bilby (*Macrotis lagotis*), Western Australia | PC |  |
|  | 99/86 | Human clinical isolate, Tasmania, Australia | PC |  |
|  | 99/87 | Human clinical isolate, Western Australia | PC |  |
|  | 99/88 | Human clinical isolate, Western Australia | PC |  |
|  | 99/89 | Human clinical isolate, Tasmania, Australia | PC |  |
|  | 99/90 | Human clinical isolate, Tasmania, Australia | PC |  |
|  | 1726 | Isolated from an armadillo (*Dasypus novemcinctus*) | ITM | [10] |
|  | 1717 | Isolated from an armadillo (*Dasypus novemcinctus*) | ITM | [10] |
|  | 2000-372 | Human clinical isolate, France | IP | [11] |
|  |  |  |  |  |
| *E. coli* | DH10B | F^-^ *endA1 recA1 galE15 galK16 nupG rpsL* Δ*lacX74* Φ*80lacZΔM15* *araD139* Δ(ara,leu)7697 *mcrA* Δ(*mrr-hsdRMS-mcrBC*) λ^-^ | Invitrogen |  |
|  | TOP10 | F^-^ *mcrA* Δ*(mrr-hsdRMS-mcrBC*) φ*80lacZΔM15* Δ*lacX74 nupG recA1 araD139* Δ(*ara-leu)7697* *galE15* *galK16* *rpsL*(Str^R^) *endA1* λ^-^ | Invitrogen |  |
|  | Rosetta 2 | F^-^ *ompT hsdS*_B_(r_B_^-^ m_B_^-^) *gal dcm* pRARE2 (Cam^R^) | Novagen |  |
|  | Rosetta-gami | Δ(*ara*-*leu*)*7697* Δ*lacX74* Δ*phoA Pvu*II *phoR araD139 ahpC galE galK rpsL* (DE3) F′[*lac^+^ lacI^q^ pro*] *gor522*::Tn*10 trxB* pLysSRARE2 (Cam^R^, Str^R^, Tet^R^) | Novagen |  |
|  | C43 | Double mutant derivative of BL21(DE3) |  | [12] |

^a^  IP, Institut Pasteur; ITM, Institute for Tropical Medicine; ATCC, American Type Culture Collection; RCH, Royal Children’s Hospital; VIDRL, Victorian Infectious Diseases Reference Laboratory; QDRLMD, Queensland Diagnostic and Reference Laboratory for Mycobacterial Diseases; ICPMR, Institute of Clinical Pathology and Medical Research; RML, NIH/NIAID/DIR Rocky Mountain Laboratories; NCTC, National Collection of Type Cultures; PC, Western Australian Centre of Pathology and Medical Research.

^b^ These strains were originally named variously as *M. pseudoshottsii*, *M. marinum,* and *M.* “liflandii”, however, in accordance with recent recommendations we have named them as *M. ulcerans* strains.

**References**

1. Stinear TP, Mve-Obiang A, Small PL, Frigui W, Pryor MJ, et al. (2004) Giant plasmid-encoded polyketide synthases produce the macrolide toxin of *Mycobacterium ulcerans*. Proc Natl Acad Sci USA 101: 1345-1349.

2. Stinear T, Ross BC, Davies JK, Marino L, Robins-Browne RM, et al. (1999) Identification and characterization of IS2404 and IS2606: two distinct repeated sequences for detection of *Mycobacterium ulcerans* by PCR. J Clin Micro 37: 1018-1023.

3. Stinear TP, Jenkin GA, Johnson PD, Davies JK (2000) Comparative genetic analysis of *Mycobacterium ulcerans* and *Mycobacterium marinum* reveals evidence of recent divergence. J Bact 182: 6322-6330.

4. Faber WR, Arias-Bouda LM, Zeegelaar JE, Kolk AH, Fonteyne PA, et al. (2000) First reported case of *Mycobacterium ulcerans* infection in a patient from China. Trans R Soc Trop Med Hyg 94: 277-279.

5. Tsukamura M, Kaneda K, Imaeda T, Mikoshiba H (1989) A taxonomic study on a mycobacterium which caused a skin ulcer in a Japanese girl and resembled *Mycobacterium ulcerans*. Kekkaku 64: 691-697.

6. Stinear T, Davies JK, Jenkin GA, Portaels F, Ross BC, et al. (2000) A simple PCR method for rapid genotype analysis of *Mycobacterium ulcerans*. J Clin Micro 38: 1482-1487.

7. Rhodes MW, Kator H, McNabb A, Deshayes C, Reyrat JM, et al. (2005) *Mycobacterium pseudoshottsii* sp. nov., a slowly growing chromogenic species isolated from Chesapeake Bay striped bass (*Morone saxatilis*). Int J Syst Evol Microbiol 55: 1139-1147.

8. Trott KA, Stacy BA, Lifland BD, Diggs HE, Harland RM, et al. (2004) Characterization of a *Mycobacterium ulcerans*-like infection in a colony of African tropical clawed frogs (*Xenopus tropicalis*). Comp Med 54: 309-317.

9. Ucko M, Colorni A, Kvitt H, Diamant A, Zlotkin A, et al. (2002) Strain variation in *Mycobacterium marinum* fish isolates. Appl Environ Microbiol 68: 5281-5287.

10. Stragier P, Ablordey A, Meyers WM, Portaels F (2005) Genotyping *Mycobacterium ulcerans* and *Mycobacterium marinum* by using mycobacterial interspersed repetitive units. J Bacteriol 187: 1639-1647.

11. Chemlal K, Huys G, Laval F, Vincent V, Savage C, et al. (2002) Characterization of an unusual Mycobacterium: a possible missing link between *Mycobacterium marinum* and *Mycobacterium ulcerans*. J Clin Microbiol 40: 2370-2380.

12. Miroux B, Walker JE (1996) Over-production of proteins in *Escherichia coli*: mutant hosts that allow synthesis of some membrane proteins and globular proteins at high levels. J Mol Biol 260: 289-298.
