# Supplementary material for: Serological Evaluation of Mycobacterium ulcerans Antigens Identified by Comparative Genomics
Source: PLoS Negl Trop Dis. 2010 Nov 2;4(11):e872. doi: 10.1371/journal.pntd.0000872 (PMC2970529; doi:10.1371/journal.pntd.0000872)
Supplement: Table S2 — Oligonucleotides used in this study. (0.08 MB DOC) [file pntd.0000872.s003.doc]

**Supplementary table 2 – Oligonucleotides used in this study**

| **CDS** | **Forward primer 5’ – 3’** | **Reverse primer 5’ – 3’** |
| --- | --- | --- |
| MUL_0027 | CACCATGGTGACCGAGGACTATGAC | GACAGCGTGCGAGAGGAC |
| MUL_0076 | CACCATGCTGCTGGCGACTTTGAC | CGATCCGGTCAGCTCTGGTC |
| MUL_0503 | CACCATGAACATGCCCGCGTTGGCCATGG | CAACTGGCGCCCAGATGGTGCGGT |
| MUL_0508 | CACCATGGCCTCTCCATCAC | ATCGTAGGTAACTCGTTCGG |
| MUL_0510 | CACCATGTATCGCGCTGTCC | TCGGTGATTCCCCCCCTTGT |
| MUL_0511 | CACCATGCTCGTGGAGGCCG | GCCCGGGCACCGGATGTCTT |
| MUL_0512 | CACCATGACCGAGCAATACGCTAAT | CTCGGCGTTGTAGAAGTCGT |
| MUL_0513 | CACCATGGCCGTACCTCTGCTTC | GAGGGTGAACAGGTTCTTCAG |
| MUL_0515 | CACCATGACCACTCCCTTGTTTCCAA | GGGTGGCTCAGGATCAACGTGT |
| MUL_0516 | CACCATGGCTTGGACTACTG | TCCGTAGGTCGGAAATTTCA |
| MUL_0517 | CACCATGGACACCGAGACC | AACCCGATCCACCGCAGGTA |
| MUL_0526 | CACCATGTCCATCCCCAAATGGCTCCAAA | TGCCGACTCCCGACTTGTCGCTGC |
| MUL_0527 | CACCATGAATTTCCTCGCCACTCTCGGCC | TTCACTGCGCTGCCCTCCGGCGGT |
| MUL_0551 | CACCATGCCGCCGTCCCTCG | ATCAACAACGCCGGAGCCCT |
| MUL_0552 | CACCATGAGCGACCACGCGG | CGCGACCTCGGGGCCGGTGA |
| MUL_0998 | CACCATGCCAAAATCAGTCCAGGT | CCACACGCTGTTCAGGTC |
| MUL_0999 | CACCATGTGGTATCCCGACCCATC | GCGGTCGAATATGTGCGTCAGTT |
| MUL_1001 | CACCATGATGTCGACCTCGGCGACCCAG | CCTAGTCTCTCTGCGGACGACGTCT |
| MUL_1135 | CACCATGGCGGGTCAGGATTTC | CTGCTCGCGACGTATTCCTTC |
| MUL_2232 | CACCATGTTGATGCGTACCG | GGCTTCTATCACCTCAGGAT |
| MUL_2590 | CACCATGAGGTGTCGGTGGCGATT | GGCCGTCCCATTTGTTCCAT |
| MUL_2831 | CACCATGTCCGCGGATCGCC | CGCCGCTATGGATAACACAA |
| MUL_2832 | CACCATGTCAGGAGTGACCA | GCCCGTCGCCTGGCGGATGT |
| MUL_3210 | CACCATGGTCGTCGATGATGTTGA | GGACACCTCGTTGGGTCGTT |
| MUL_3212 | CACCATGAGTCAAACGACCG | CGCAGCCGCGCGCATCATAA |
| MUL_3214 | CACCATGGGCCTGCCTAGCT | TGCGGCGAGCAGCAAGTCCG |
| MUL_3215 | CACCATGAACCTGGAGGTCCG | CGTCAGCTCCGCAAGCACTA |
| MUL_3216 | CACCATGGGCACTTCAGGCT | CGGCGTGCCTCCGCCGCGTG |
| MUL_3217 | CACCATGGCGGAGGAACTCG | ATTAGTACCTCCATCGAGC |
| MUL_3218 | CACCATGGGTCGAGTCCGTGTCGCTGCG | AGCACGGCGCTGTTCCCAATCC |
| MUL_3230 | CACCATGGGGGTGAAGATCG | AGTGGTCGCAGGTTCAAATC |
| MUL_3440 | CACCATGTCGGTGATCGACC | GCTGATCACCGACACGGTGT |
| MUL_3828 | CACCATGCGCGACCGGCGTG | CACTCCGGCCCCAATAGCGC |
| MUL_4213 | CACCATGTTCAATCGGTGCGAGTT | TTACACCTGGGGTGCATTC |
| MUL_4217 | CACCATGGGATTTCTTGTCGAGTC | GCGACCAAACCGCGCGCCCCC |
| MUP002 | CACCATGACAAACGCGCCCG | CGCTGACAGCAGTTCGGTG |
| MUP003 | CACCATGTCACGAAAGTGG | CGGTGGTGGGCCGAAGTCGG |
| MUP004 | CACCATGAGCAAGTTAACGC | CGCGTCCTTCCTGCCGGGCA |
| MUP006 | CACCATGAGCACGCAGCTAA | CTTGGCCTTGCCGTGCGGCC |
| MUP007 | CACCATGAACGCAATCCAGC | TGCGCCGCAAGCTCTCGCCG |
| MUP013 | CACCATGCGAACAAGTCGCGTTCTCGTC | CCGAGTGGAACTCAAGGTCCTCGTC |
| MUP014 | CACCATGTTGATCTCGGGCTTGTGGGCAC | CCGGCCTACAGTCGCTGTTACCGGC |
| MUP015 | CACCATGGCTCGCTGGCCAGCCCACCCGCGCC | ACACCGCTGCCTGCTCTCGGTAGCC |
| MUP016 | CACCATGATGATGGCCGCGT | TAAGGTGTGCAGCCTGTGCA |
| MUP017 | CACCATGTCACATCCGTACACCAACG | CGTCCCACCTGTGTTGTAGCC |
| MUP018 | CACCATGCAACAGCCCACAG | CGCCTGACTCAGCAGTGATG |
| MUP019 | CACCATGCTCGGTATCACAT | CGCGACATTTTGGGCGACCT |
| MUP020 | CACCATGTCCACTGACTCAC | TAGCGAGCACCATCCTTGAA |
| MUP021 | CACCATGACCGCGGCGAACC | CGCGTGCTCGATAACTCGAC |
| MUP023 | CACCATGAGCCCACGGCCCA | CGCGATGCGGTAGCCGATCT |
| MUP024 | CACCATGGGTGGATCGACTG | CCAGCGCGCGAGATCTGCTA |
| MUP038 | CACCATGATTGTTTGGCCCG | CGCGGCCGCGAGATCGACAT |
| MUP045 | CACCATGATTTGGAATGACA | CTACGAAGTGGAGTGTCCGG |
| MUP046 | CACCATGGGTTGGCGTTGGT | TACCGACGAAAACCCACCAG |
| MUP057 | CACCATGCAATCAACGACTGCCTCCGTCCCGG | GCCTTCGCACAGCGCAAGTCCGCGC |
| MUP064 | CACCATGCCTCCGCCAGGTTGGTAT | GAGTGCAAGCGCCAGGAT |
| MUP065 | CACCATGTACCTACGCGGTG | GCCACGAGCCTGGGAAGCGT |
| MUP066 | CACATGCCAACCGCTGTAG | CCACAGCGTCGGCATATCCC |
| MUP067 | CACCATGTACCCCGGCTGTG | GCCGGCATGGCTGCGCTGAA |
| MUP068 | CACCATGTATGGGTGGTTCCGGCCGGCGGGGA | CACGGGGTGGGTCTCAGACAAGGTA |
| MUP070 | CACCATGTCCCGATACCCGA | TCTTCGTGGTTTTGTGATGG |
| MUP071 | CACCATGCTTCGATCCCCCG | CGATCCGCAACCGCGATGTG |
| MUP074 | CACCATGAGCGTTGTTCTGCATCC | GGCTGCGTTGACCCTGTCCT |
| MUP075 | CACCATGCAAGCAGATGAAA | TATGCTCACCGCCGGATGCG |
| MUP076 | CACCATGAATCCGCAGCCAGTGGGCCAGC | CACACACGCCTCGTCGAGTTGCCTG |
| MUP078 | CACCATGCTACACGTCGACA | CGCGATTTGTCTGGCTCTTT |
| MUP079 | CGCGATTTGTCTGGCTCTTT | GAAATCAATCAGGGCTTCAT |
| MUP080 | CACCATGCGTTGGGCGATCA | CGCCGTCGAGATGGTCAATG |
| DH | CACCATGGATTTCAGCGGGG | AGGCCACGGCACAGGCGTTA |
| KRB | CACCATGGAGGGCACGGTGTTGAT | GTAGTCGGCGAGAGCATCTAA |
| KRA | CACCATGAGGGGGACCGTGTTAATC | GGCGAGCGAATCTAAGAATG |
| AT-Ac-I | CACCATGCGTCTGTACCAGCATCTCA | ATAGTCAGGTGAGGCGAGTTG |
| AT-P | CACCATGGCAGTGGGTGTACTGGTG | CATTGTGGTGGTGTCGTAGG |
| ER | CACCATGCTTGACACCACCGGCAAG | CATGTCACGTAAGGCCGAGAT |
| ACP1 | CACCATGCACACCCCCGAAAGCATCA | TGTGGGGTGATCGAAGATAAG |
| ACP2 | CACCATGGATCAAGCCGCATCCGTT | GGGTGTGGGGTGATCGAA |
| ACP3 | CACCATGCACACCCCCGAAAGCATC | GGTGTGCAGGTGTTGGGTTAG |
| KS | CACCATGCAGTTGTGGGACTTGGTG | GGTTAGGAGTCGGACTGTGC |
| KS load | CACCATGAATTTAGACGGCCTCGTT | GCCACCCATGGAAAAAGATGA |
| AT-Ac-II | CACCATGTTTGTGCCCTGGGTGATT | GTGGTCCACCCAGTAACCTG |
|  |  |  |
|  |  |  |
