# Supplementary material for: Serological Evaluation of Mycobacterium ulcerans Antigens Identified by Comparative Genomics
Source: PLoS Negl Trop Dis. 2010 Nov 2;4(11):e872. doi: 10.1371/journal.pntd.0000872 (PMC2970529; doi:10.1371/journal.pntd.0000872)
Supplement: Table S3 — M. ulcerans genes tested in this study. (0.06 MB DOC) [file pntd.0000872.s004.doc]

**Supplementary table 3 – *M. ulcerans* genes tested** in this study

| **CDS** | **Predicted size (kDa)** | **Predicted Product Function** | **Putative domains** | **Expressed** |  |
| --- | --- | --- | --- | --- | --- |
| MUL_0512 | 52.6 | Hypothetical | None identified | C-term 6xHis |  |
| MUL_0513 | 28.3 | Hypothetical | None identified | C-term 6xHis |  |
| MUL_0515 | 58.1 | Hypothetical | None identified | C-term 6xHis |  |
| MUL_0516 | 32.4 | Hypothetical | None identified | N-term 6xHis |  |
| MUL_0998 | 7.1 | Hypothetical | None identified | N-term 6xHis |  |
| MUL_0999 | 14.1 | Hypothetical | 3 TMDs | N-term 6xHis |  |
| MUL_1001 | 41.1 | Hypothetical | 2 TMDs | C-term 6xHis |  |
| MUL_2831 | 10.4 | Hypothetical | None identified | N-term 6xHis |  |
| MUL_3212 | 17.7 | Hypothetical | None identified | C-term 6xHis |  |
| MUL_3216 | 30.6 | Hypothetical | None identified | N-term 6xHis |  |
| MUL_3217 | 58.5 | P-loop ATPase | KAP family P-loop domain (pfam07693) | N-term/C-term 6xHis |  |
| MUL_3218 | 25.7 | Metalloprotease | Predicted metal-dependent hydrolase (pfam01863) | C-term 6xHis |  |
| MUL_5129 | 59.7 | Prophage integrase | Serine recombinase catalytic domain (pfam07508) | N-term 6xHis |  |
| MUP002 | 11.5 | Hypothetical | None identified | N-term 6xHis |  |
| MUP003 | 22.4 | Hypothetical | None identified | C-term 6xHis |  |
| MUP004 | 21.8 | Hypothetical | None identified | Not cloned |  |
| MUP013 | 16.1 | Hypothetical | 2 TMDs | None |  |
| MUP014 | 26.9 | Hypothetical | 5 TMDs | None |  |
| MUP015 | 28.9 | Hypothetical | Predicted signal sequence | N-term/C-term 6xHis |  |
| MUP016 | 28.0 | Hypothetical | Predicted signal sequence | None |  |
| MUP017 | 16.7 | Hypothetical | 1 TMD | None |  |
| MUP019 | 36.2 | Hypothetical | 1 TMD | None |  |
| MUP023 | 23.4 | Hypothetical | None identified | C-term 6xHis |  |
| MUP024 | 20.6 | Hypothetical | None identified | N-term 6xHis |  |
| MUP038 | 32.7 | Type II thioesterase | Lysophospholipase (COG2267) | C-term 6xHis |  |
| MUP045 | 34.7 | B-keto acyl synthetase | 3-oxoacyl-ACP synthase III (PF08541.3) | C-term 6xHis |  |
| MUP057 | 27.1 | Hypothetical lipoprotein | None identified | C-term 6xHis |  |
| MUP064 | 12.6 | Hypothetical | 3 TMDs | N-term 6xHis |  |
| MUP068 | 38.2 | Hypothetical | 1 TMD | N-term/C-term 6xHis |  |
| MUP074 | 20.1 | Hypothetical | 1 TMD | None |  |
| MUP076 | 23.0 | Hypothetical | 1 TMD | None |  |
| KS core | 39.0 | Ketosynthase | Ketosynthase | C-term 6xHis |  |
| KS alt | 39.3 | Ketosynthase | Ketosynthase | C-term 6xHis |  |
| DH dom | 15.8 | Dehydratase | Dehydratase | C-term 6xHis |  |
| At acetate 1 | 34.0 | Acyltransferase | Acyltransferase | C-term 6xHis |  |
| At acetate 2 | 34.5 | Acyltransferase | Acyltransferase | C-term 6xHis |  |
| AT propionate | 35.9 | Acyltransferase | Acyltransferase | C-term 6xHis |  |
| ER | 31.4 | Enoylreductase | Enoylreductase | C-term 6xHis |  |
| KR A | 17.2 | Ketoreductase | Ketoreductase | N-term 6xHis |  |
| KR B | 16.5 | Ketoreductase | Ketoreductase | N-term 6xHis |  |
| ACP 1 | 4.9 | Acyl carrier protein | Acyl carrier protein | C-term 6xHis |  |
| ACP 2 | 6.0 | Acyl carrier protein | Acyl carrier protein | N-term 6xHis |  |
| ACP 3 | 6.0 | Acyl carrier protein | Acyl carrier protein | C-term 6xHis |  |
| MUL_2232 (*hsp18*, *acr3*) | 16.5 | Molecular chaperone, small Hsp family | Hsp20/alpha crystallin family (pfam00011) | C-term 6xHis |  |
| MUL_1393 (*hsp65*, *groEL2*) | 56.5 | Molecular chaperone, GroEL2 | TCP-1/cpn60 chaperonin family (pF00118.17) | C-term 6xHis |  |
